# Cytokines and oral cancer risk: Genetic evidence from a bidirectional Mendelian randomization study

**DOI:** 10.1097/MD.0000000000042642

**Published:** 2025-06-06

**Authors:** Wenbin Shi, Anan Zhang, Yuli Xu, Shuhua Liu, Xiqun Jia, Ziyang Hu

**Affiliations:** a Department of Stomatology, Shenzhen Longhua District Central Hospital, Shenzhen, China; b Department of Neonatal, Shenzhen Longhua District Central Hospital, Shenzhen, China.

**Keywords:** cytokine, genome-wide association study, Mendelian randomization, oral cancer, SNPs

## Abstract

This study aimed to elucidate the causal relationship between cytokines and oral cancer using Mendelian randomization (MR) analysis. Utilizing genetic data from genome-wide association studies (GWAS) and publicly available datasets, we conducted a bidirectional 2-sample MR analysis. The study design employed single nucleotide polymorphisms as genetic instruments to investigate the link between cytokines and oral cancer. The analysis was based on data from 2 cohorts with total 132 cytokines: 41 cytokines from comprehensive GWAS meta-analysis data, 91 cytokines from GWAS summary statistics for circulating inflammatory cytokines. Oral cancer genetic association data was sourced from the FinGen R10 datasets. To discern the causal relationship between cytokines and oral cancer, 5 MR methodologies, including inverse variance weighted and MR-Egger regression, weighted median, weighted mode, and simple mode were applied. The MR analysis revealed nominal associations between certain cytokines and the risk of oral cancer. Specifically, increased levels of C-X-C motif chemokine ligand 9 (odd ratios [OR] = 0.760, 0.600–0.962, 95% confidence interval [CI] 0.600–0.962, *P* = .023), monocyte chemoattractant protein 1 (OR = 0.78, 95% CI 0.32–0.99, *P* = .046), and TNF related activation induced cytokine (OR = 0.792, 95% CI 0.630–0.994, *P* = .044) were associated with a reduced risk of oral cancer, while higher levels of monocyte chemoattractant protein 2 (OR = 1.164, 95% CI 1.001–1.353, *P* = .048) and CC motif chemokine 25 (OR = 1.434, 95% CI 1.106–1.858, *P* = .006) were linked to an increased risk. The reverse analysis suggested a possible effect of oral cancer on the level of circulating cytokines, particularly Fractalkine (OR = 0.942, 95% CI 0.897–0.990, *P* = .019). No evidence of heterogeneity or significant pleiotropy was detected, validating the instrumental variables used. The findings support a causal relationship between specific cytokines and the risk of oral cancer, highlighting the complex interplay between inflammatory mediators and cancer development. These results underscore the importance of individualized immune profiling in treating oral cancer patients and pave the way for future research into targeted therapies based on cytokine profiles.

## 1. Introduction

Oral cancers, predominantly oral squamous cell carcinomas, constitute a significant global health burden and present a complex challenge in the field of oral medicine. Arising from squamous epithelial cells, these cancers form a major segment of head and neck squamous cell carcinoma, ranking as the seventh most prevalent cancer worldwide. Ghantous et al^[1]^ note that oral cancer comprise the majority of oral and oropharyngeal cancers. Sung et al’s interpretation of GLOBOCAN 2020 data^[2]^ sheds light on the severity of this issue, indicating that oral cancer represent 2.5% of all cancer cases and account for 2% of cancer deaths globally. This data highlights the critical need for intensive research and the development of effective management approaches in addressing oral cancer.

Patients with oral cancer exhibit a distinctive cytokine profile, characterized by the elevated expression of cytokines including IL-6, IL-10, TNFα, C-X-C motif chemokine ligand 9 (CXCL9), and monocyte chemoattractant protein 1 (MCP-1), as evidenced in studies.^[3,4]^ This cytokine signature mirrors patterns observed in peripheral blood and plays a crucial role in the tumor microenvironment. These cytokines not only modify immune cell behavior but also potentially promote cancer cell growth amidst immune surveillance. For example, elevated levels of IL-6 in oral and oropharyngeal cancer patients correlate with the deactivation of the p53 tumor suppressor gene.^[5]^ The presence of TGF-β and IL-10 within the tumor microenvironment underscores the complex interplay that fosters chronic inflammation, aiding in cancer cell proliferation and metastasis.^[6,7]^ Furthermore, cytokines like CXCL9 and MCP-1 are implicated in steering neutrophils towards a pro-tumoral phenotype, thereby complicating the immune response in oral cancer scenarios.^[6,8–10]^ These insights underline the pivotal influence of cytokines on tumor advancement and the immune response, underscoring the necessity for individualized immune profiling in treating oral and oropharyngeal cancer patients. Nevertheless, there remains a notable gap in our understanding of plasma cytokines and immune checkpoint proteins in oral cancer patients.

Mendelian randomization (MR), an epidemiologic method employing genetic variants such as single nucleotide polymorphisms (SNPs) as instrumental variables (IVs), forms the foundation of our study. This approach is instrumental in elucidating potential causal relationships between various exposures and outcomes.^[11]^ The advantage of MR lies in its reliance on the random allocation of inherited variants during gamete formation, which aids in circumventing biases from potential confounding factors and reversing causality.^[12,13]^ While recent research has applied MR to understand the causal links between oral cancer and numerous risk factors,^[14,15]^ the causal relationship between cytokines and oral cancer remains unexplored. Our study is thus designed to utilize MR to determine whether a causal connection exists between cytokines and oral cancer.

To achieve this, we have utilized genetic data from genome-wide association studies (GWAS) and publicly available datasets. This has enabled us to perform the first comprehensive bidirectional 2-sample MR analysis, a pioneering effort aimed at systematically investigating the possible causal relationship between cytokines and the risk of oral cancer.

## 2. Materials and methods

### 2.1. Study design

**Figure 1.**
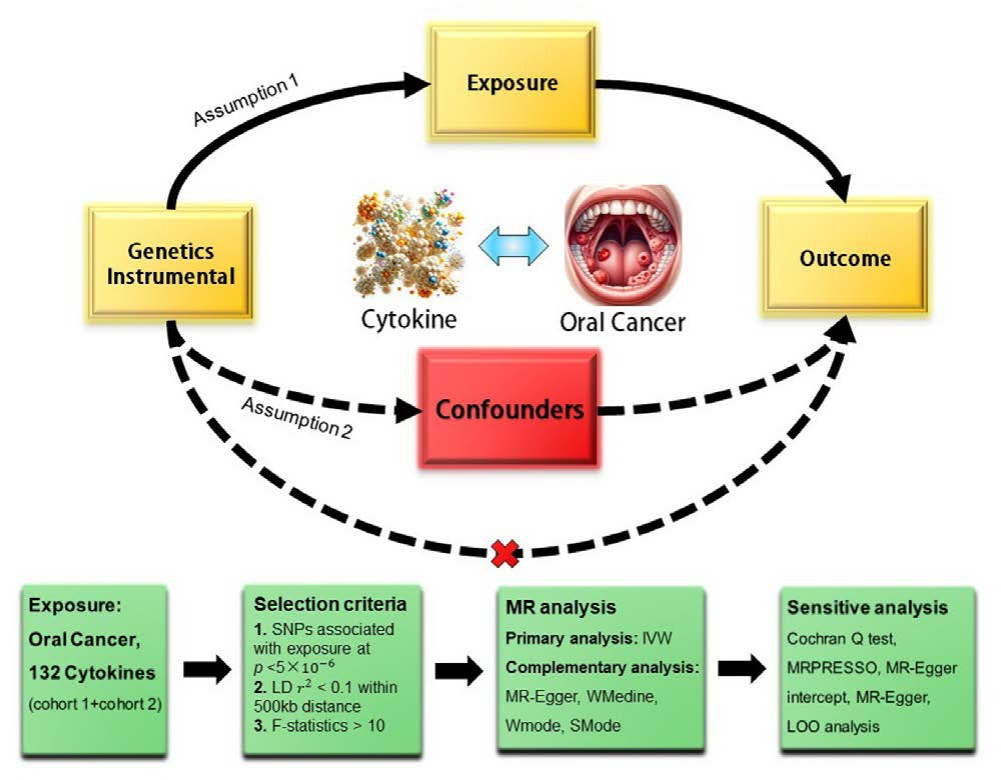
Diagram of the bidirectional MR analysis. Assumption 1, genetic instruments are strongly associated with the exposures (inflammatory of interest); Assumption 2, genetic instruments are independent of confounding factors; Assumption 3, genetic instruments are not associated with outcome and affect outcome (oral cancer) only via exposures. Two cohorts of cytokines were included in this study. IVW = inverse variance weighted, LD = linkage disequilibrium, LOO analysis = leave-one-out analysis, MR = Mendelian randomization, MR-PRESSO = MR-Pleiotropy RESidual sum and outlier, SMode = simple mode, SNPs = single nucleotide polymorphisms, WMedine = weighted median, WM = weighted mode.

This research, conducted as a bidirectional 2-sample MR study, rigorously explores the link between cytokines and oral cancer, employing SNPs as a genetic instrument. Figure 1 delineates the study’s design framework. The integrity and effectiveness of the MR analysis hinge on 3 critical assumptions: the IVs must exhibit a robust connection with the exposure variable; these IVs should maintain independence from confounders that might impact both exposure and outcome; the influence of IVs on the outcome should be exclusively mediated through the exposure.^[16]^ In our study, the initial assumption underwent thorough scrutiny during the IV selection process. The latter 2 assumptions, inherently unverifiable empirically, were rigorously evaluated using sensitivity analyses and systematic assessments for pleiotropy by the research team (Fig. 1). Adherence to the STROBE-MR guidelines, which bolster the reporting standards of observational studies in epidemiology utilizing MR, was a cornerstone of our methodology. This study was approved by the Ethics Committee of our Hospital (2024-021-01).

### 2.2. Cytokines data source

To analyze the up to date causal relationship between cytokines and oral cancer. There are 2 cohort of cytokines included in this study (Fig. 1).

Cohort 1: The first cohort utilized comprehensive GWAS meta-analysis data, focusing on the circulating levels of 41 cytokines, with a total participant count of 8293. This cohort comprised genomic and cytokine information from 4608 individuals from the FINRISK 1997 study and an additional 1705 participants from FINRISK 2002. We quantified cytokines by analyzing EDTA-treated plasma from FINRISK 1997, heparinized plasma from FINRISK 2002, and serum samples from the YFS study. To mitigate confounding variables such as age, sex, BMI, and genetic differences, we meticulously adjusted the genetic associations. Cytokine measurements were conducted using the Bio-Plex 200 system, supported by Bio-Plex 6.0 software, with a comprehensive summary of GWAS cytokine data presented in Table S1, Supplemental Digital Content, https://links.lww.com/MD/P73 for MR analysis.

Cohort 2: The second cohort was derived from the SCALLOP Consortium’s latest research, which provided GWAS summary statistics for circulating inflammatory cytokines. This analysis included 91 proteins, using the Olink Target-96 Inflammation panel, with modifications due to BDNF limitations (Table S2, Supplemental Digital Content, https://links.lww.com/MD/P73).^[17]^ The data generation was executed at Olink’s laboratories in Uppsala, employing SNP arrays for genotyping, followed by imputation using either the 1000 Genomes or HRC panel. A GWAS analysis for each protein in the pQTL mapping was conducted, aggregating data from 14,824 participants and establishing a statistical significance threshold of *P* ≤ 5 × 10^−10^.^[17]^ This study’s findings were juxtaposed with those of the ARISTOTLE study, with clear definitions of pQTLs that reflect associations with protein abundance.^[18,19]^ Protein variance was calculated using a specific formula, and conditional analysis was performed using GCTA.^[17]^

### 2.3. Oral cancer data source

The genetic association data for oral cancer originated from the FinGen’s study. The R10 version included in this study is up-to-data publicly available for FinGen’s dataset. The FinnGen study is an extensive research project in Finland that combines genomic data from 4,12,181 participants (2,30,310 females and 1,81,871 males), integrates various national health registers for detailed phenotyping. It uses Finnish versions of international classification systems for disease endpoints, and manual selection processes for the most representative endpoints. Genotyping was performed using Illumina and Affymetrix arrays, with stringent quality control measures, and the data was aligned to the latest reference genome.

### 2.4. Instrument selection

In the process of selecting genetic instruments, SNPs were identified based on their significant and independent associations with cytokines in cohort 1 and cohort 2 in the primary analysis. Initial calculations revealed that, following the removal of redundant variables and clumping, only 11 out of the 41 cytokines had more than 3 SNPs, using a stringent significance threshold (*P* < 5 × 10^−8^). However, this number was deemed insufficient for comprehensive pleiotropic analysis. Consequently, to ensure a sufficient number of instruments for the MR analysis, we adopted a more lenient significance threshold (*P* < 5 × 10^−^⁶), a strategy that has been used in similar MR studies involving cytokines.^[20–22]^ This study incorporated SNPs from previous GWAS relevant to circulating inflammatory cytokines for MR analysis, ensuring compliance with a genome-wide significance level of *P* < 5 × 10^−6^. The selection of SNPs was meticulous, with a focus on ensuring no linkage disequilibrium with other SNPs, maintaining an *r*^2^ value below 0.001 within a 10,000 kb radius, adhering to methodologies commonly employed in previous studies.^[20–22]^ To mitigate potential biases from inadequate instruments, the *R*^2^ and *F*-statistics for each SNP were calculated using specific formulas.


$$\begin{array}{l} R^{2}=\frac{2\times \beta^{2}\times EAF\times \left(1-EAF\right)}{\left[2\times \beta^{2}\times EAF\times \left(1-EAF\right)+2\times (se{\left(\beta^{2}\right)}^{2}\times N\times EAF\times \left(1-EAF\right)\right]} \end{array}$$

$$\begin{array}{l} F=\frac{N-k-1}{k}\times \frac{R^{2}}{1-R^{2}} \end{array}$$

These formulas incorporate genetic variant effect size (β), effect allele frequency, standard error of effect size (*se*[β]), exposure sample size (*N*), and the number of SNPs (*k*). SNPs with an *F*-statistic below 10 were excluded from the analysis. The study’s procedures meticulously extracted and harmonized outcome-associated SNPs, systematically excluding SNPs that were correlated (*P* < 5 × 10^−6^), palindromic, or showed allele inconsistencies, thereby enabling a robust MR analysis on cytokines supported by more than 2 SNPs.^[23]^

### 2.5. Mendelian randomization analysis

In this research, we employed bidirectional MR to investigate the influence of cytokines on oral cancer, and oral cancer on cytokines. Specifically, we examined: effect of (41 cytokines) on oral cancer, and corresponding reverse analysis; effect of cohort 2 (91 cytokines) on oral cancer, and corresponding reverse analysis. This comprehensive research utilized a variety of techniques, including inverse variance weighted (IVW), MR-Egger, weighted median, weighted mode, and simple model strategies. The overarching influence of cytokines on oral cancer was assessed through a meta-analytic approach, integrating Wald estimates for each SNP using the IVW method. In scenarios devoid of horizontal pleiotropy, IVW results are considered unbiased.^[24]^ The MR-Egger regression, which relies on the InSIDE assumption that instrument strength is independent of direct effects, evaluates pleiotropy via its intercept term; a zero intercept in MR-Egger regression aligns with IVW results, suggesting an absence of horizontal pleiotropy.^[25]^ Furthermore, the weighted median approach is capable of yielding reliable causal estimates even when up to 50% of IVs are invalid.^[26]^ In cases where the InSIDE assumption is violated, the weighted mode estimate is preferable, offering increased power, reduced bias, and a lower type I error rate compared to MR-Egger regression.^[27]^ Although the simple mode method may lack precision, it generally exhibits reduced bias compared to other methods.^[27]^ Lastly, the MR-PRESSO technique is employed to identify and adjust for horizontal pleiotropy by removing significant outliers. However, the efficacy of the MR-PRESSO outlier test hinges on the adherence to InSIDE premises and requires that the majority of genetic markers be valid instruments.^[28]^

### 2.6. Statistical analysis

All statistical analyses were executed using R (version 4.3.3), leveraging the “TwoSampleMR” and “MRPRESSO” packages for MR studies.^[29]^ To validate the significance of our findings, we employed meta-analytical techniques to conduct heterogeneity and horizontal pleiotropy tests, utilizing the modified Cochran *Q* statistic, MR-PRESSO and MR-Egger intercept to assess deviation.^[30]^ Moreover, the Steiger test was executed to counteract potential biases from reverse causality.^[31]^ The causal inference direction could be erroneous if the explained variance of IVs in oral cancer surpasses that of cytokines. In cases of binary outcomes, the causal effect was quantified as the per standard deviation increase in biomarker levels, with the results presented alongside a 95% confidence interval (CI).

## 3. Result

### 3.1. Causal effect of inflammatory cytokines on risk of oral cancer

**Figure 2.**
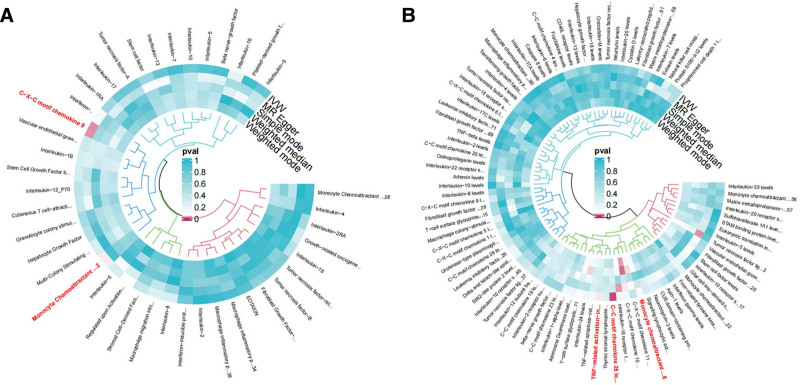
Preliminary MR analyses for the associations between cytokines and the risk of oral cancer. The circle from the outer to the inner represented the IVW, MR-Egger, simple mode weighted median, and weighted mode, respectively. The shades of color were reflections of the magnitude of the *P*-value as the label inside the circle. IVW = inverse variance weighted, MR = Mendelian randomization, MR-Egger = MR-Egger regression.

**Figure 3.**
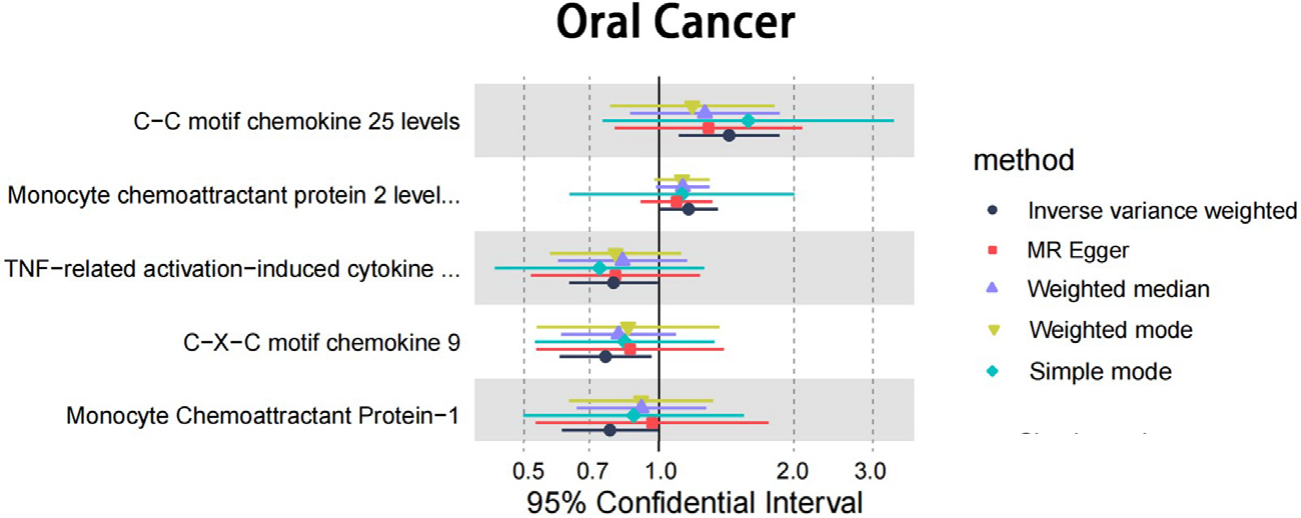
Forest plot of cytokines on the risk of oral cancer. Forest plot to visualize the causal effect of cytokines on the risk of oral cancer risk factors by inverse variance weighted, MR-Egger regression, weighted median and weighted mode method and simple mode. CCL25 and MCP-2 may increase the risk of oral cancer. TRANCE, CXCL9 and MCP-1 may decrease the risk of oral cancer. CCL25 = CC motif chemokine 25, CXCL9 = C-X-C motif chemokine ligand 9, MCP-1 = monocyte chemoattractant protein 1, MCP-2 = monocyte chemoattractant protein 2, TRANCE = TNF related activation induced cytokine.

**Figure 4.**
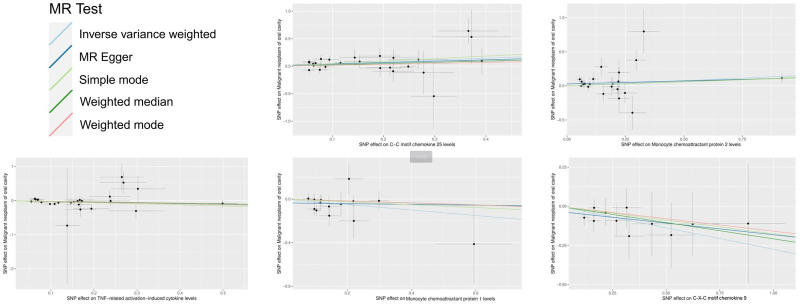
Scatter plots of cytokines on the risk of oral cancer. Analyses were conducted using IVW, MR-Egger, weighted median and weighted mode. The slope of each line corresponding to the estimated MR effect per method. IVW = inverse variance weighted, MR = Mendelian randomization, MR-Egger = MR-Egger regression.

After excluding unmatched SNPs and finding proxies in outcome data, 132 cytokines in 2 cohorts were employed in the MR analysis. These IVs comprised SNPs ranging from 11 to 27 (with CXCL9 genetically represented by 11 SNPs and TNF related activation induced cytokine (TRANCE) levels having the highest representation with 27 SNPs). *F*-statistics ranged from 20.85 to 4502.70, indicating no potential bias derived from weak instrument (Table S3, Supplemental Digital Content, https://links.lww.com/MD/P73). Statistical result for MR analysis is shown on Table S4, Supplemental Digital Content, https://links.lww.com/MD/P73. For main MR analysis employing IVW. However, nominal associations with oral cancer were observed in CXCL9 (CXCL9 → oral cancer: OR = 0.760 95% CI 0.600–0.962, 95% CI 0.600–0.962, *P* = .023), MCP-1 (MCP-1 → oral cancer: OR = 0.777, 95% CI 0.607–0.995, *P* = .046), and Monocyte chemoattractant protein 2 (MCP-2) levels (MCP-2 → oral cancer: OR = 1.164, 95% CI 1.001–1.353, *P* = .048), CC motif chemokine (CCL) 25 (CCL25 → oral cancer: OR = 1.434, 95% CI 1.106–1.858, *P* = .006). TRANCE levels (TRANCE → oral cancer: OR = 0.792, 95% CI 0.630–0.994, *P* = .044; Fig. 2 and Table 1). The analysis found no evidence of heterogeneity among the SNPs for CXCL9, MCP-1, MCP-2, CCL25, and TRANCE as indicated by Cochran’s *Q* test. Furthermore, both the MR-Egger intercept and MR-PRESSO tests showed no significant pleiotropy, reinforcing the validity of our IVs (Table 2). Directionality was evaluated using Steiger test and revealed no reverse casual direction (Table S5, Supplemental Digital Content, https://links.lww.com/MD/P73). The forest plot from 5 different MR methods are shown in Figure 3. Leave-one-out analysis affirmed that no individual SNP introduced bias into the MR estimation (Figure S1, Supplemental Digital Content, https://links.lww.com/MD/P74). Scatter plots for identified cytokine across various tests are displayed on Figure 4. The funnel plots were showed on Figure S2, Supplemental Digital Content, https://links.lww.com/MD/P74. This multi-faceted approach ensured a thorough examination of the potential causal relationships between cytokine levels and oral cancer risk, providing a solid foundation for future research in this area.

**Table 1:** IVW result of cytokines on oral cancer in 2 cohorts.

Cohort	Exposure	NSNP	B	SE	*P*-value	OR (95% CI)
1	MCP-1	14	−0.252	0.126	.046	0.777 (0.607–0.995)
1	CXCL9	11	−0.275	0.121	.023	0.76 (0.6–0.962)
2	TRANCE levels	27	−0.234	0.116	.044	0.792 (0.63–0.994)
2	MCP-2 levels	21	0.152	0.077	.048	1.164 (1.001–1.353)
2	CCL25 levels	26	0.36	0.132	.006	1.434 (1.106–1.858)

CCL25 = CC motif chemokine 25, CXCL9 = C-X-C motif chemokine ligand 9, MCP-1 = monocyte chemoattractant protein 1, MCP-2 = monocyte chemoattractant protein 2, TRANCE = TNF related activation induced cytokine.

**Table 2:** Sensitivity analysis of cytokines on oral cancer.

Cohort	Exposure	IVW Cochran	MR-PRESSO	Egger intercept
1	MCP-1	0.64	0.65	0.45
1	CXCL9	0.99	0.99	0.57
2	TRANCE levels	0.60	0.63	0.96
2	MCP-2 levels	0.15	0.30	0.27
2	CCL25 levels	0.47	0.17	0.61

CCL25 = CC motif chemokine 25, CXCL9 = C-X-C motif chemokine ligand 9, MCP-1 = monocyte chemoattractant protein 1, MCP-2 = monocyte chemoattractant protein 2, TRANCE = TNF related activation induced cytokine.

### 3.2. Causal effect of risk of oral cancer on inflammatory cytokines

**Figure 5.**
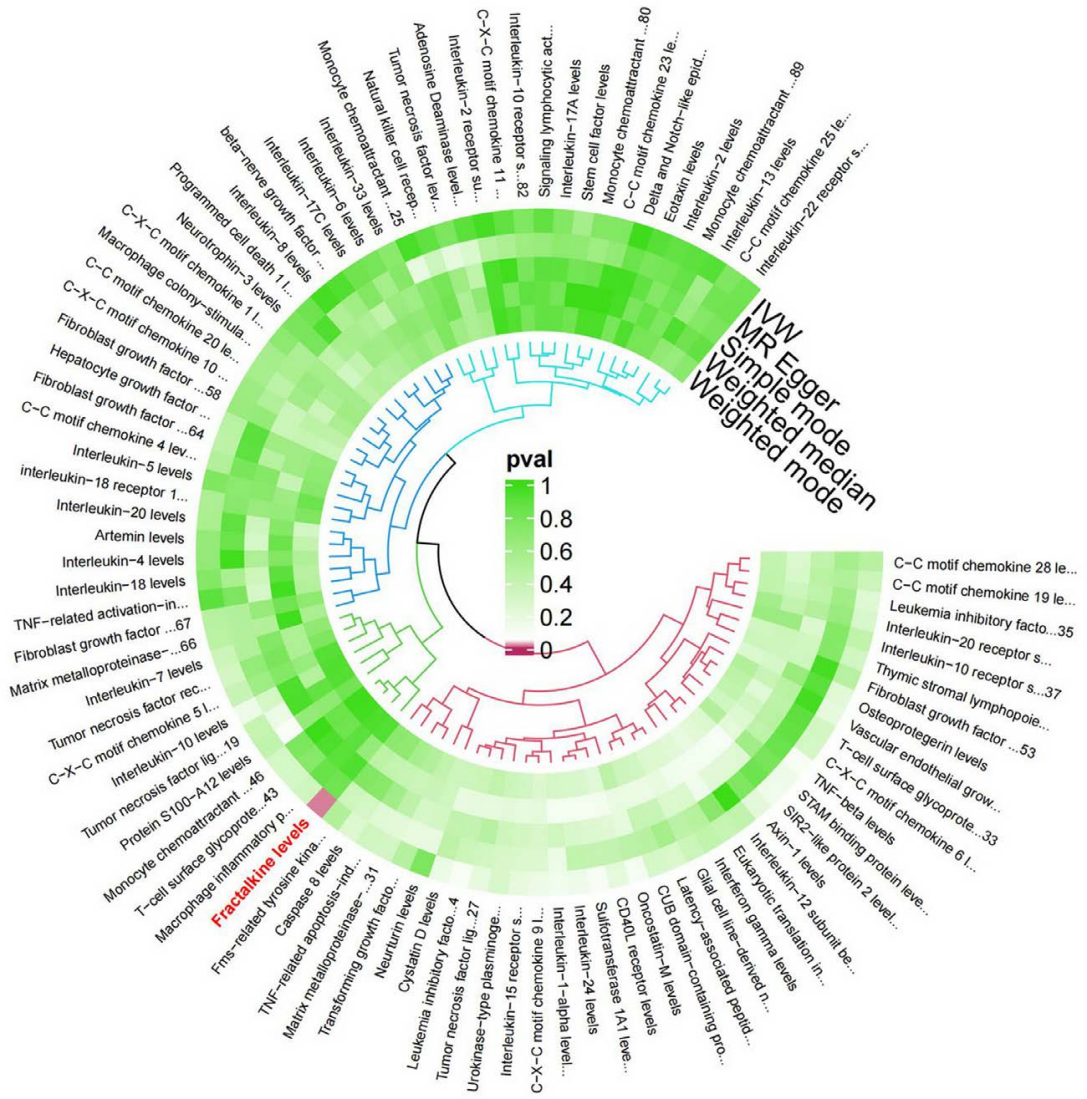
Preliminary MR reverse analyses for the associations between cytokines and the risk of oral cancer. The circle from the outer to the inner represented the IVW, MR-Egger, simple mode weighted median, and weighted mode, respectively. The shades of color were reflections of the magnitude of the *P*-value as the label inside the circle. IVW = inverse variance weighted, MR = Mendelian randomization, MR-Egger = MR-Egger regression.

**Figure 6.**
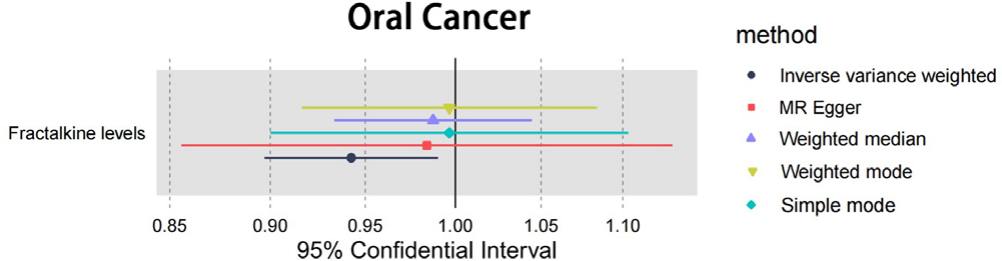
Forest plot of oral cancer on the risk of cytokines. Forest plot to visualize the causal effect of oral cancer on the risk of cytokines by inverse variance weighted, MR-Egger regression, weighted median and weighted mode method and simple mode. Oral cancer may increase the risk of Fractalkine levels.

In the reverse analysis, a total of 8 SNPs were selected as instrument. No evidence of weak instrument was identified, with *F*-statistic> 10 (Table S6, Supplemental Digital Content, https://links.lww.com/MD/P73). Analytical results by 5 methods are shown in Table S7, Supplemental Digital Content, https://links.lww.com/MD/P73. However, there were suggestive effects of oral cancer (oral cancer → Fractalkine: OR = 0.942, 95% CI 0.897–0.990, *P* = .019) on circulating cytokines (Fig. 5 and Table 3). The forest plot from 5 different MR methods are shown in Figure 6. Sensitivity analyses did not reveal significantly detrimental results for exposures of interest (Table 4, Table S8, Supplemental Digital Content, https://links.lww.com/MD/P73). Scatter plots for identified cytokine across various tests are displayed on Figure S3, Supplemental Digital Content, https://links.lww.com/MD/P74.

**Table 3:** IVW result of oral cancer on cytokines.

Outcome	NSNP	B	SE	*P*-value	OR (95%CI)
Fractalkine levels	8	−0.059	0.025	.019	0.942 (0.897–0.990)

**Table 4:** Sensitivity analysis of oral cancer on cytokines.

Outcome	IVW Cochran	MR-PRESSO	Egger intercept
Fractalkine levels	0.07	0.09	0.54

## 4. Discussion

Numerous observational researches has established a correlation between the concentrations of circulating cytokines and the incidence of oral cancer. However, such studies often grapple with biases stemming from limited sample sizes and the presence of confounding variables, which may distort the outcomes. Furthermore, the domain lacks robust genetic evidence to corroborate this correlation. In response, we harnessed the most recent GWAS data and employed a comprehensive analytical strategy to investigate the causative influence of 132 distinct cytokines on oral cancer. Departing from conventional MR investigations, our approach was not confined to individual cytokines. Instead, we conducted a bidirectional MR analysis, a novel method that assesses the mutual causative link between 2 variables and elucidates the direction of causality. This technique enables the identification of cytokines that play a role either before or subsequent to the disease mechanism. Additionally, we leveraged various MR methodologies to bolster the study’s rigor and mitigate the impact of pleiotropic effects. We initially considered using a stringent *P*-value threshold (*P* < 5 × 10^−^⁸) for SNP selection, but this resulted in too few SNPs for a robust MR analysis. Therefore, we chose a more lenient threshold (*P* < 5 × 10⁻⁶), commonly used in cytokine-related MR studies,^[20–22]^ which strikes a balance between instrument strength and statistical power. This threshold, supported by existing literature, allows for the inclusion of SNPs with weaker but still meaningful associations with cytokine levels. Sensitivity analyses confirmed that this approach did not introduce bias and ensured the robustness of our findings. The aim of this research was to methodically evaluate the potential causative association between 2 cohorts of cytokines and the risk of oral cancer through bidirectional MR analysis, thereby providing a more nuanced understanding of their role in the etiology of the disease.

MCP-1, also known as CCL2, monocyte chemotactic activating factor, is the product of the human JE gene and a member of the family of C-C (or β) chemokines MCP-1 is a potent basophil activator but does not affect eosinophils. In our study, the observed association (OR = 0.78, 95% CI 0.32–0.99, *P* = .046) between CCL2 levels and oral cancer suggests a nuanced, protective role of CCL2 against oral cancer, highlighting the complex interplay of cytokines in cancer progression. Our findings align with existing literature that identifies chemokines like CCL2 as crucial in regulating tumor behavior, potentially serving as biomarkers or therapeutic targets in oral cancer.^[9,10]^ The study underlines the reduced serum CCL2 levels in OSCC patients compared to healthy individuals, suggesting its potential utility in OSCC diagnosis and indicating the significance of cytokine profiling in understanding OSCC’s pathogenesis. Moreover, the variability in CCL3 expression and its association with tumor size highlight the heterogeneity of the immune response in OSCC and underline the potential of CCL2 and CCL3 as biomarkers for disease progression and prognosis.^[9]^ Our study also contributes to the understanding of the CCL2-CCR2 axis in the context of lymphatic metastasis, corroborating previous findings on the presence of CCL2 in tumor-associated neutrophils and its expression in moderately or poorly differentiated SCC at primary tumor sites.^[32]^ Moreover, the observed infiltration of CCR2-positive macrophages and mesenchymal cells within carcinoma nests and particularly in the marginal sinus of metastatic cases underscores the axis’s significance in the metastatic process.^[32]^

The role of MCP-2, also known as CCL8 alongside other chemokines like CCL2 and CCL7, in recruiting immune cells to the tumor microenvironment and influencing cancer cell proliferation and metastasis, offers a nuanced understanding of oral cancer’s pathogenesis.^[33]^ Our study unveils the significant role of CCL8 in the progression of oral cancer with a direct correlation established between CCL8 levels and oral cancer risk (OR = 1.164, 95% CI 1.001–1.353, *P* = .048). Oral cancer increasingly prevalent in regions like Sri Lanka due to cultural practices such as betel quid chewing. In Shafana et al’s study, CCL8 has been highlighted as a tumor suppressor gene alongside TAGLN2 and CCND2.^[34]^ The capacity of CCL8 to influence immune cell recruitment and cancer cell behaviors underlines its critical role in the disease’s pathogenesis.^[33,34]^ Our findings highlight the chemokine’s potential as a biomarker for early detection and a target for novel treatment strategies. Among the critical findings of this study is the identification of CCL8 as a key marker gene associated with oral cancer.

Our study demonstrated that increased CCL25 levels are associated with a higher odds ratio (OR = 1.434) for oral cancer, suggesting a pro-tumorigenic effect. Although direct research on the relationship between CCL25 and oral cancer is still lacking, our study results, along with related literature from other areas of cancer research, underscore the significance of CCL25 in tumor progression.^[35,36]^ CCL25’s interaction with CCR9 has been shown to suppress apoptosis in cancer cells by activating the PI3K/AKT pathway, leading to the survival and proliferation of tumor cells.^[33]^ This antiapoptotic effect, highlighted in lung and breast cancer studies, may partly explain the association between elevated CCL25 levels and an increased risk of oral cancer observed in our study. Furthermore, CCL25 has been demonstrated to promote tumor cell proliferation and facilitate metastasis by enhancing the migratory and invasive potential of cancer cells toward CCL25 gradients.^[35,37,38]^ This pro-metastatic role of CCL25, documented in cancers such as melanoma and hepatocellular carcinoma, supports the notion that CCL25 could contribute to oral cancer progression through similar mechanisms.

In our study examining the impact of cytokines on oral cancer, we observed findings regarding CXCL9 and TRANCE that differ from their roles in previous study. Regarding CXCL9, a chemokine that typically attracts T cells and NK cells to the tumor site to promote a protective immune response.^[39]^ Our study also find an inverse association between CXCL9 levels and oral cancer risk, OR of 0.760 (95% CI 0.600–0.962, *P* = .023), suggesting a protective effect of CXCL9 against OSCC. CXCL9 enhances antitumor immunity by recruiting immune cells through CXCR3, inhibiting angiogenesis to limit tumor growth, and promoting tumor cell migration and metastasis through direct interaction with CXCR3-expressing cells.^[40]^ While elevated serum CXCL9 indicates tumor aggressiveness, it also suggests a systemic antitumor response.^[40]^ Chang et al’s study demonstrated elevated CXCL9 in OSCC tissues and serum correlates with advanced disease and poorer survival (OR for higher disease stages and worse survival outcomes), underscoring CXCL9’s complex role.^[8]^ The suppression of CXCL9 in cellular models attenuates cancer cell proliferation and invasion, reinforcing its multifaceted role in OSCC. Thus, CXCL9 may have both protective and tumor-promoting effects depending on the situation. Moreover, TRANCE, also known as receptor activator of NF-κB ligand, plays a significant role in modulating immune responses. Our study found that TRANCE may protect against oral cancer (OR 0.792, 95% CI 0.630–0.994, *P* = .044). TRANCE binds to the RANK receptor on dendritic cells, promoting their maturation and enhancing tumor antigen presentation to T cells, which strengthens the adaptive immune response. This process indirectly activates cytotoxic T lymphocytes, crucial for targeting and destroying tumor cells.^[41]^ However, its involvement in the NF-κB signaling pathway also suggests that TRANCE could facilitate cancer progression in some contexts, which contrasts with its role in bone resorption and tumor growth in diseases like multiple myeloma.^[42]^ These insights highlight CXCL9 and TRANCE as a nuanced prognostic marker, emphasizing the need for further research to fully understand its therapeutic potential in oral cancer.

The study also provide important insights into how oral cancer influences the cytokine profile, highlighting a bidirectional interaction between the tumor and its microenvironment. Incorporating our MR study findings, oral cancer is associated with a slight reduction in Fractalkine levels (OR = 0.942, 95% CI 0.897–0.990, *P* = .019), suggesting that oral cancer may influence Fractalkine levels negatively. This insight underscores the complexity of Fractalkine’s role in OSCC, highlighting its involvement in processes such as bone invasion and cell migration through the TGF-β regulation and the CX3CL1-CX3CR1 signaling pathway.^[43,44]^ Fractalkine regulates the recruitment of immune cells such as monocytes, T cells, and natural killer cells through its receptor CX3CR1.^[45]^ A decrease in Fractalkine levels may be related to the recruitment of these immune cells to the tumor site, weakening immune surveillance and the antitumor immune response.^[45]^ However, the effectiveness of Fractalkine as a marker for perineural invasion remains in question due to the lack of significant association found in studies, pointing to the intricate interplay between oral cancer and Fractalkine levels.^[46]^ This contrast emphasizes the nuanced but critical role of Fractalkine in OSCC’s pathogenesis and progression, indicating the need for further research to fully understand its potential as a biomarker and therapeutic target, especially considering its varied effects across different aspects of OSCC pathology, such as PNI detection. The OR value and associated literature highlight a complex relationship between oral cancer and Fractalkine, warranting more investigation to clarify its clinical implications.

Our study goes beyond identifying correlations by demonstrating that specific cytokines, such as CCL2 and CCL8, have a causal role in oral cancer risk, revealing mechanisms behind cancer development. This highlights how chronic inflammation drives progression and suggests potential therapeutic targets. The bidirectional approach is a key innovation, examining both the impact of cytokines on oral cancer and the reverse effect of cancer on cytokine levels, providing a more comprehensive view of immune dynamics in the tumor microenvironment. Additionally, by exploring 132 cytokines, our study fills a critical knowledge gap and offers a broader understanding of the cytokine landscape in oral cancer. The findings from our study also have translational potential. For diagnostic platforms: The cytokine profiles identified in our study could serve as biomarkers for early detection of oral cancer. For example, cytokines like CCL2 and CCL8 could be used in blood or saliva-based diagnostic tests. These biomarkers could be incorporated into high-throughput screening platforms for noninvasive and cost-effective early detection of oral cancer, which is crucial for improving patient outcomes through earlier intervention. Therapeutic Targets: Our identification of cytokines such as CCL2 and CCL8 as key drivers of oral cancer progression presents opportunities for therapeutic targeting. Inhibiting the CCL2-CCR2 axis could help modulate immune responses, slowing down tumor growth and metastasis. Furthermore, targeting CXCL9 and TRANCE levels could enhance the effectiveness of existing cancer immunotherapies. This could lead to more personalized and effective treatments for oral cancer patients.

There are several limitations existing within this study. Firstly, the scope of the genome-wide exploration was constrained by a finite array of SNPs. To address this, a marginally more lenient threshold was adopted for MR analysis, aligning with protocols observed in preceding studies. Despite these adjustments, the strength of our IVs is bolstered by *F*-statistic values for the selected SNPs exceeding 10, underscoring their robustness. Secondly, the investigation’s reliance on GWAS data exclusively sourced from individuals of European descent narrows the spectrum of ethnic diversity, highlighting the imperative for further empirical scrutiny to extend the pertinence of these insights to a broader demographic range. This necessitates future validation efforts. Thirdly, the precision of MR estimates is closely tied to the size of the sample population, accentuating the critical need for an enlargement of the study cohort to enhance the credibility of the findings. Fourthly, due to the reliance on publicly available GWAS data, which is limited to a specific population (Finnish), we were unable to conduct laboratory experiments such as cell-based assays or cytokine level measurements in biological samples. These experiments would require access to clinical cohorts, ethical approval, and substantial resources, which were not feasible within the scope of this study. However, our findings provide a foundation for future experimental studies. Laboratory-based validation using cell models, animal studies, or clinical samples will be necessary to confirm the causal relationships observed in this MR study and to further explore the mechanisms underlying the associations between cytokines and oral cancer.

## 5. Conclusion

The findings support a causal relationship between specific cytokines and the risk of oral cancer, highlighting the complex interplay between inflammatory mediators and cancer development. These results underscore the importance of individualized immune profiling in treating oral cancer patients and pave the way for future research into targeted therapies based on cytokine profiles.

## Acknowledgments

We express our gratitude to all SCALLOP Consortium and FinnGen studies, QTLbase working group, GWAS Catalog for providing open access to the summary association statistics data.

## Author contributions

**Conceptualization:** Ziyang Hu.

**Data curation:** Anan Zhang, Yuli Xu, Shuhua Liu, Xiqun Jia.

**Formal analysis:** Wenbin Shi, Anan Zhang, Yuli Xu, Shuhua Liu.

**Investigation:** Shuhua Liu, Xiqun Jia, Ziyang Hu.

**Methodology:** Shuhua Liu, Xiqun Jia, Ziyang Hu.

**Project administration:** Xiqun Jia, Ziyang Hu.

**Software:** Wenbin Shi, Anan Zhang.

**Supervision:** Xiqun Jia.

**Validation:** Wenbin Shi, Yuli Xu, Xiqun Jia, Ziyang Hu.

**Writing – original draft:** Wenbin Shi.

## Supplementary Material


