# Supplementary material for: Cytokines and oral cancer risk: Genetic evidence from a bidirectional Mendelian randomization study
Source: Medicine (Baltimore). 2025 Jun 6;104(23):e42642. doi: 10.1097/MD.0000000000042642 (PMC12150936; doi:10.1097/MD.0000000000042642)

Figure S1 Leave-one-out analysis of cytokines on the risk of oral cancer

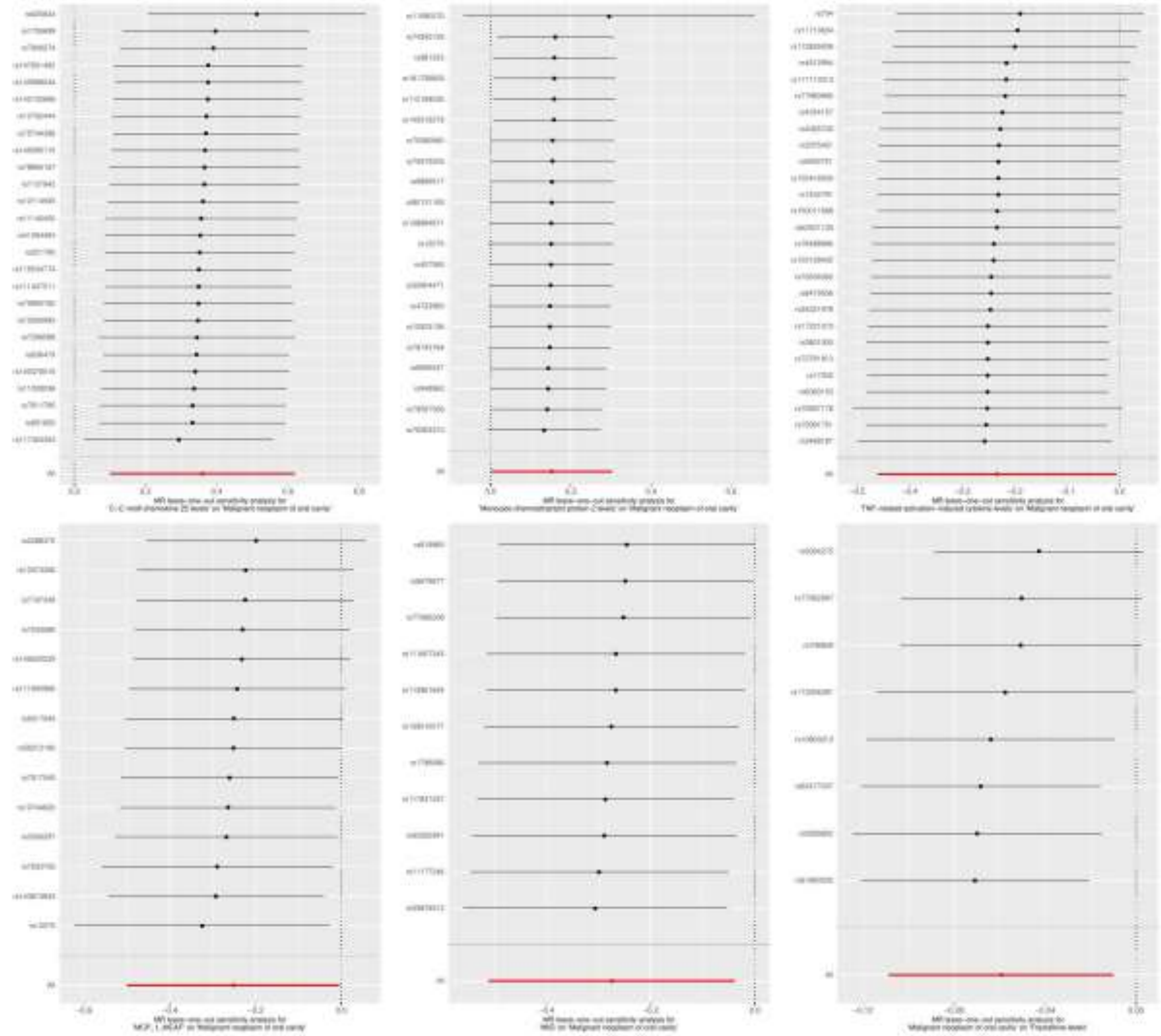

Figure S2 Funnel plots of cytokines on the risk of oral cancer

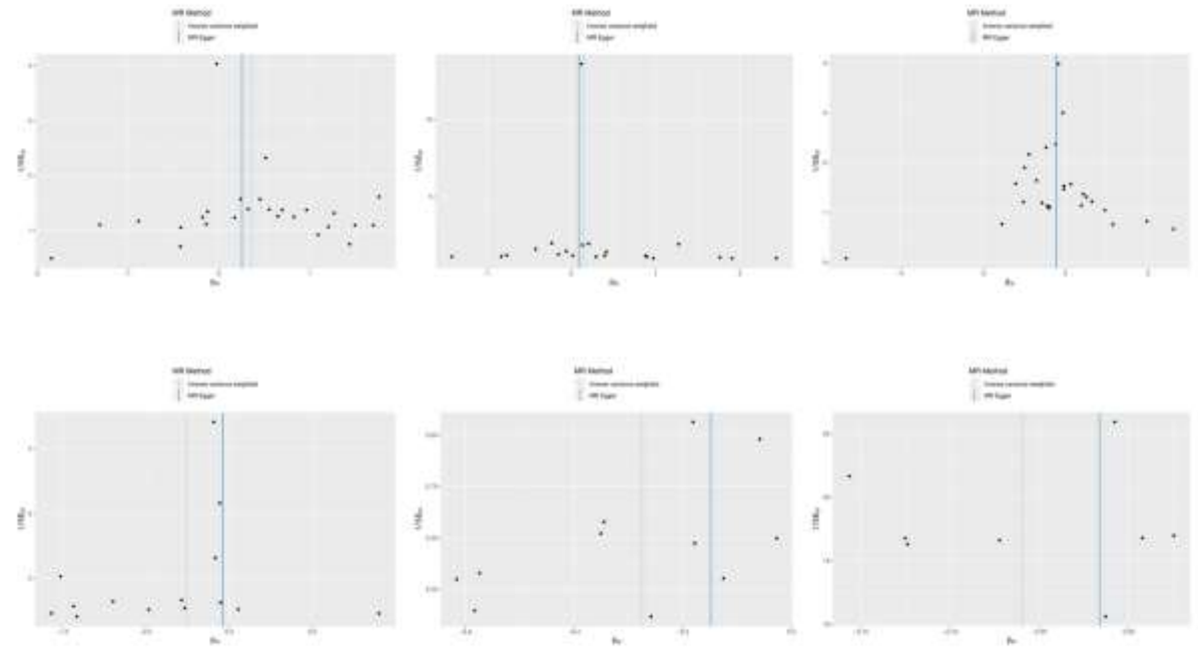

Figure S3 Scatter plots of oral cancer on the risk of cytokines

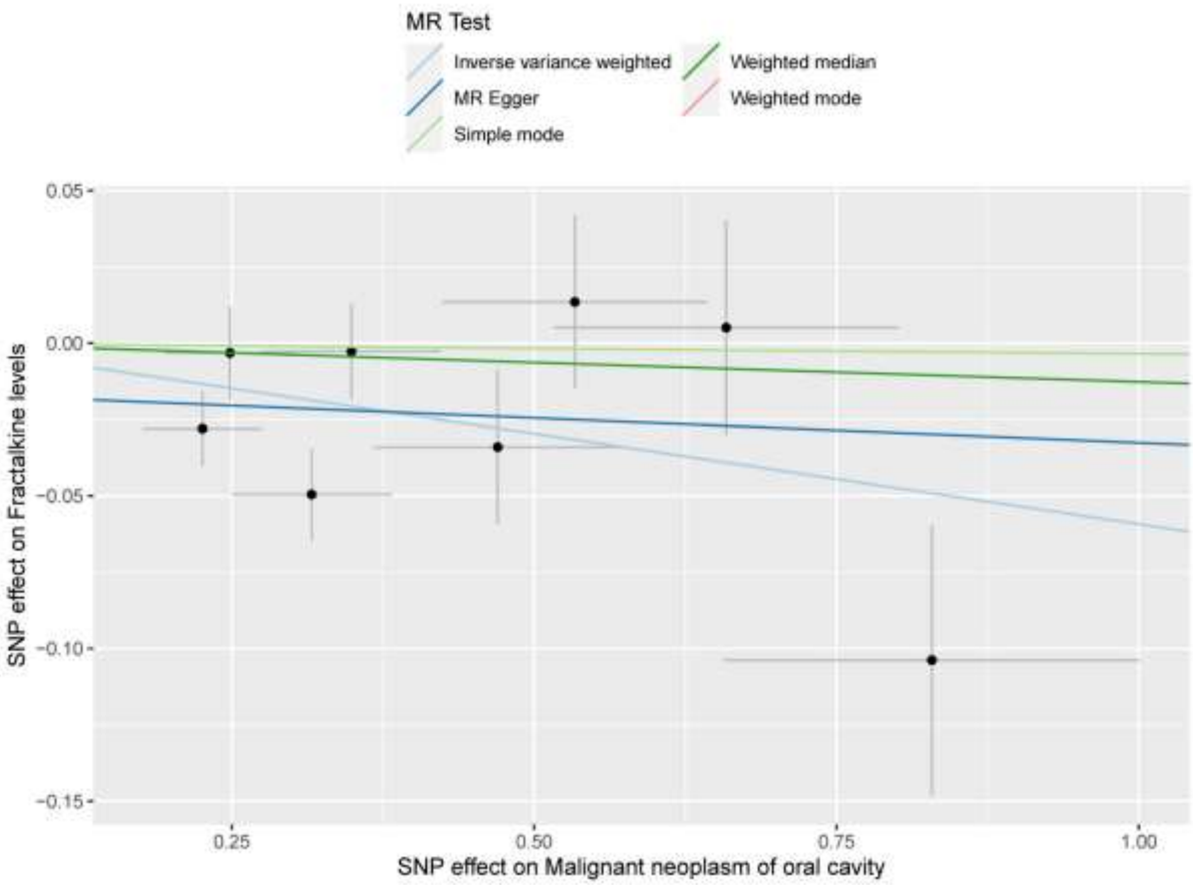

Supplement: Supplementary file 2 [file medi-104-e42642-s002.pdf]
